# Editorial: Application of fishes as biological models in genetic studies

**DOI:** 10.3389/fgene.2022.1092160

**Published:** 2023-01-05

**Authors:** Tony Silveira, Sandra Isabel Moreno Abril, Caroline Gomes Lucas, Mariana Härter Remião

**Affiliations:** ^1^ Biological Sciences Institute, Federal University of Rio Grande, Rio Grande, Brazil; ^2^ Marine Research Centre, University of Vigo, Vigo, Spain; ^3^ Department of Ecology and Animal Biology, University of Vigo, Vigo, Spain; ^4^ Division of Animal Sciences, Animal Science Research Center, National Swine Resource and Research Center, University of Missouri, Columbia, MO, United States; ^5^ Structural Genomics Laboratory, Technological Developmental Center, Federal University of Pelotas, Pelotas, Brazil

**Keywords:** evolution, biotechnology, genomics, aquaculture, chromosomal diversity, microsatellites, microRNA, transcriptome

## Introduction

Although Gregor Mendel is considered the father of genetics, while alive, he never took credit for his principles on heredity. Mendel’s treatises have been part of the collections of the largest European libraries since the 19th century but were apparently forgotten by science until their rediscovery in 1900 (Watson et al., 2013). Mendel’s revolutionary ideas could have possibly given greater strength to the formulation of Charles Darwin’s ideas regarding common descent and gradual evolution through natural selection, as presented in “The Origin of Species”. Nonetheless, Darwin envisioned the possibility of genetic heredity by describing “invisible characters” emerging in atavistic situations and naming his hypothetical particles of heredity as “gemmules” (Darwin, 1868). It is remarkable that the “invisible characters” and “gemmules” referred to by Darwin are what we now know as genes—a term coined in 1909 by Wilhelm Johannsen.

During the 1930s and 40’s, the findings of great proponents of genetics and evolution such as Mendel, Darwin, Wallace, Fisher, Haldane, Wright, Dobzhansky, Mayr, and others were brought together to form the neo-Darwinian synthesis (Mayr, 2004). In addition, during the 40’s, a molecular revolution began in genetics, which, driven by sequencing technology, gave rise to the late 70’s genomics era. It took approximately 100 years to formulate the theoretical foundations of genetics in order to understand how information is transmitted from generation to generation. Now, less than 45 years after the beginning of the genomic era, it is possible to identify complete genomes in less than a week, as evidenced by the COVID-19 pandemic (Virological.org, 2020 ^1^).

The comparative use of *in vivo* models such as mice, rats, zebrafish, and fruit flies have been fundamental for the advancement of medicine and biological research. Among animals, fishes fall between the most relevant groups in genetic studies, due to their diversity and plasticity. Although fish studies played a role in applying and corroborating Mendel’s findings in the first few decades of the 19th century, these studies contributed little to the development of classical genetics. However, fish studies have been of great importance for the development of modern molecular genetics and other “omics” sciences.

Due to this increase in genomic knowledge, several fish species have become important biological models around the world. For example, zebrafish have been used as avatars of human patients in personalized medicine (Usai et al., 2020), tilapia islets have been studied in the treatment of diabetes (Wright et al., 2014), and tilapia skin has been studied for the healing of burn lesions (Costa et al., 2019; Dias et al., 2020). Moreover, fish can be used for the study of genes and genomes, epigenetics, and genetic expression, as presented in the articles of this Research Topic. The increase in knowledge and the importance of fishes in scientific research would certainly not be possible without studies like these. Together, they provide a glimpse of the *Application of Fishes as Biological Models in Genetic Studies* around the globe.

The broad diversity of the studies submitted in this Research Topic is organized into sub-topics that are listed below, but in fact, many of the articles fall under more than one sub-topic. This highlights the nowadays multifaceted character of the application of fishes in genetic studies.

## Fishes as genetic models

The *Austrolebias* genus, a group of annual killifish, has been proposed by García et al. as an excellent model for evolutionary genomic processes due to its large genome size—probably associated with transposable elements—as well as its high chromosome instability, the occurrence of natural hybridization between sister species, and burst speciation events.

Another suggestion of fish as an animal model is *Astyanax altiparanae*, as reviewed by Yasui et al. This species, popularly known as yellowtrail tetra, can be used as an alternative for studies directed to neotropical fishes. *A. altiparanae* has been considered as the most advanced fish within the Neotropical region regarding fish biotechnology. It has already been used to describe artificial fertilization, germ cell transplantation, chromosome set manipulation, and other technologies with applications in aquaculture and conservation of genetic resources.

Beck et al. suggest that the threespine stickleback (*Gasterosteus aculeatus*) can be a model for human diseases related to mitochondrial DNA. In this study, the first complete coding region analysis of the two mitotypes of *G. aculeatus* is provided, from which, mitogenomic divergence can be extended to other mammal models including humans. Dohi and Matsui reinforce, in a brief research report, that small fish can be used for prospective studies on human aging, since genes involved in human pathways and diseases are shared with various fish species such as zebrafish, medaka, and the turquoise killifish (*Nothobranchius furzeri*).

## Fishes as models in evolutionary studies

Genetic and molecular studies are also performed on fish species to clarify evolutionary aspects of the species. Fernandes et al. have applied conventional cytogenetic procedures and fluorescence *in situ* hybridization of 18S rDNA, 5S rDNA, H3, and H2B-H2A histone sequences in *Acanthurus* species. The authors describe the relations and evolutionary differentiation among *A. coeruleus*, *A. chirurgus*, and *A. bahianus* populations inhabiting coastal regions of the Southwest Atlantic, South Atlantic oceanic islands, Greater Caribbean, and Indo-Pacific Ocean.

In a similar manner, Silva et al. A) have performed a comparative study on two *Peckoltia* species and found that both species present 2n = 52 karyotypic formulas, although their chromosomal bands and the repetitive chromosomal sites between them were different. The same research group published another study on this topic, but with a comparison of two *Ancistrus* species from the Amazon region. Silva et al. B) found extensive chromosomal diversity between both analyzed species, presenting different karyotypes with distinct patterns of organization.

Through mitochondrial DNA sequencing and nuclear DNA microsatellites, Astorga et al. present the genetic diversity and divergence between landlocked and migratory populations of *Galaxias maculatus*, distributed in southern Chile. Additionally, the genetic diversity was significantly higher in migratory populations than in landlocked populations, presenting a higher differentiation among lakes than estuaries.

Using cytogenetic studies on *Gymnotus carapo*, Machado et al. hypothesize that it is not a single widespread species, but a group of cryptic species. Chromosome painting showed more complex rearrangements, a high number of repetitive DNA sites, and extensive karyotype reorganization in comparison with previous studies that used classical cytogenetics.

## Fishes as models in biotechnology

In-depth knowledge of the fish genome can provide new alternatives to overcome old barriers. Merlano et al. present the production of recombinant cystatin-B (rCYST-B) from red piranha *Pigcentrus nattereri* to control bacterial growth in aquaculture. This protein exhibited bacteriostatic action, inhibiting *Escherichia coli* and *Bacillus subtilis* growth, suggesting its potential biotechnological use.

## Molecular biology for aquaculture improvement

For Nile tilapia, two studies in this Research Topic have brought new clarifications in the production and aquaculture of this species, using a molecular approach. Martins et al. evaluate the expressions of genes responsible for appetite regulation, metabolic and physiological changes, and osmoregulation in tilapia that were exposed to different salinity concentrations. Gene modulation generated by a salinity increase may have contributed to a decrease in weight gain and growth rate, as well as an increase in oxidative damage to blood cells.

Chen et al., also regarding Nile tilapia, demonstrate that individuals with higher growth rates have significantly higher total protein, total triglyceride, total cholesterol, and high- and low-density lipoproteins, but significantly lower glucose levels when compared with individuals in the lower growth rate group. Transcriptomics has also been evaluated, showing that more than 1,000 genes were differentially expressed between the higher and lower growth rate groups.

## Fishes as models in genomics

Graciano et al. have presented the first *de novo* genomic assembly for *Salminus brasiliensis*, one of the most important species for angling and consumption in southern South America. This study also presented the coding genome annotations of 12,962 putative genes from assembled genomic fragments over 10 kb as well as a genome-wide panel for predicted microsatellites.

The discovery of molecular markers is very important in fish studies, as they can be employed for the detection of adulteration and authenticity (Kotsanopoulos et al., 2021), toxicological research (Santos et al., 2018; Kar et al., 2021), conservation (Tan et al., 2019; Bourret et al., 2020), and phylogenetic studies (Saad, 2019; Prabhu et al., 2020). In this Research Topic, Goes et al. have characterized three new satellitomes from three species: *Psalidodon fasciatus*, *P. bockmanni*, and *Astyanax lacustris*. Finally, Pagano et al. have shown that the microRNA miR-429 can be differentially expressed in *Odontesthes humensis*, a possible biomarker for osmotic stress.

## Conclusion

In summary, this Research Topic presents new advances in molecular studies and the proposal of fishes of different species as biological models in order to understand the evolution and distribution of species, to produce new molecules for biotechnological applications, and to optimize aquaculture and conservation of species. Fishes are gaining more importance in scientific research. Therefore, we hope this topic could instigate the readers to look for new questions and find new answers, walking in the Mendel and Darwin’s footsteps and going beyond guided by knowledge that the fishes brought us after them.

## Footnotes

1
https://virological.org/t/first-cases-of-coronavirus-disease-covid-19-in-brazil-south-america-2-genomes-3rd-march-2020/409.
